# Are published standards for haematological indices in pregnancy applicable across populations: an evaluation in healthy pregnant Jamaican women

**DOI:** 10.1186/1471-2393-8-8

**Published:** 2008-02-28

**Authors:** Tameika R James, Harvey L Reid, Anthony M Mullings

**Affiliations:** 1 Department of Basic Medical Sciences, Physiology Section, Faculty of Medical Sciences, UWI, Mona Campus, Kingston 7, Jamaica, West Indies; 2 Department of Basic Medical Sciences, Physiology Section, Faculty of Medical Sciences, UWI, Mona Campus, Kingston 7, Jamaica, West Indies; 3 Department of Obstetrics, Gynaecology and Child Health, Faculty of Medical Sciences, University of the West Indies, Mona Campus, Kingston 7, Jamaica, West Indies

## Abstract

**Background:**

The haematological profile of the pregnant woman has an impact on the outcome of the pregnancy. Published guidelines indicate acceptable levels for haematological indices in pregnancy but they are population specific. Indicators of haemoglobin concentration are the most commonly utilized of the indices. These published international norms are used across populations, however, there is no evidence confirming their applicability to a population such as the Jamaican pregnant woman. This study was therefore undertaken with the intent of documenting the haematological profile of pregnant primigravid Jamaican women and comparing these to the established norms to determine whether the norms apply or whether there was a need to establish local norms.

**Methods:**

This was a longitudinal study done on a cohort of 157 healthy primigravid women ages 15 to 25 and without anaemia, and who were recruited from the antenatal clinic of the University Hospital of the West Indies, Kingston, Jamaica. The haemoglobin concentration, packed cell volume, mean cell volume, mean cell haemoglobin, mean cell haemoglobin concentration, white blood cell count, red blood cell count and platelet count were measured on samples of blood obtained from each consenting participant during each of the three trimesters. The results were analysed using SPSS for windows (Version 11) and the data expressed as means ± S.D. Means were compared using the student's paired *t-test*. Comparison was then made with the international norms as recommended by the United States Center for Disease Control (1989). Ethical approval for this study was obtained from the University Hospital of the West Indies/University of the West Indies Ethics Committee.

**Results:**

The results showed changes by trimester in all measured variables. For most of the indices the changes achieved levels of significance across trimesters. These changes were however in keeping with the expected physiological response in pregnancy and the values were similar to the published international norms.

**Conclusion:**

The findings suggest that the international norms for haematological indices in pregnancy are applicable across populations and to the pregnant Jamaican primigravid woman. This finding may be reassuring to others with a similar population and stage of development as Jamaica.

## Background

The haematological profile of an individual to a large extent reflects their general health [1] and many studies have identified the haematological profile of the pregnant woman as one of the factors affecting pregnancy and its outcome [2-7]. The most commonly referred to of the haematological indices are the indicators of haemoglobin concentration, and low haemoglobin (anaemia) is the most widely identified haematological abnormality [8] and is associated with adverse pregnancy outcome [3-5]. Anaemia in women is variously defined with the two most common being either as a haemoglobin concentration less than 11.0 g/dl or <5th percentile of the distribution of haemoglobin concentration or Haematocrit in a healthy reference population and is based on age, sex, and (among pregnant women) stage of pregnancy. According to the World Health Organisation, "anaemia is a common and serious problem in pregnancy" and needs to be addressed [9].

Since the assessment of haematological status is possible through a series of tests measuring different variables it is valuable to have norms for the haematological indices. Although profiles are generally available in the published literature as established norms [8], there is no evidence that any studies were done to assess the applicability of these norms across populations and especially to the Jamaican primigravid patient. Such a study is of importance since antenatal care and pregnancy outcome is in part, predicated on the monitoring of and response to these haematological indices [2-9]. It is therefore the intent of this study to investigate the haematological profile of pregnant primigravid Jamaican women and compare these to the established norms to determine whether the norms apply or if there is a need to establish local norms. We believe that this information could also be of benefit to other countries with a similar population and stage of development as Jamaica.

## Methods

The definition of anaemia as used in this study was a haemoglobin of <11.5 g/dl. The results are also compared with the finding if the definition of <5^th ^percentile, quoted above, was used. One hundred and fifty seven (157) primigravid women between the ages of 15 and 25 were recruited on their first visit to the antenatal clinic at the University Hospital of the West Indies. The first visit is usually made at 9–12 weeks gestation. Both oral and written consent was obtained from each participant. The Inclusion criteria included age 15 to 25, primigravida status, haemoglobin 11.0 g/dl or greater, and no history of chronic illness. Exclusion criteria included age younger than 15 and older than 25, not primigravid, history of anaemia or chronic illness. The age limit of 15 to 25 was chosen as it was felt they are more likely to represent a healthy segment of the pregnant population. After enrolment in the study, 5 ml of venous blood was drawn and anticoagulated with K^+ ^EDTA at a concentration of 1.5 mg/ml. Blood samples were again obtained at 26 – 28 and 36 – 38 weeks gestational age. The blood samples were analyzed for haemoglobin (Hb), packed cell volume (PCV), mean corpuscular haemoglobin (MCH), mean corpuscular haemoglobin concentration (MCHC), mean corpuscular volume (MCV), red blood cells (RBC), platelets (Plt) and white blood cells (WBC) by means of an automated Coulter Counter A^c^.T diff™ Analyzer. All measurements were made within 2 hours of Venepuncture. Data analysis was done using SPSS for windows (Version 11) and the data expressed as means ± S.D. Means were compared using the student's paired *t-test*. Comparison was made with the international reference levels as well as with the 5^th ^percentile for the study group. Ethical approval for this study was obtained from the University Hospital of the West Indies/University of the West Indies Faculty of Medical Sciences Ethics Committee.

## Results

### General characteristics of the cohort

Table 1 shows the demographic profile of the participants. The average age was 20.31 ± 2.33 yrs, while average height was 162.70 ± 7.43 cm. The average weight of the participants increased progressively over the three trimesters of pregnancy.

**Table 1:** Demographic characteristics of participants

Age group (yrs)		Age (yrs)	Height (cm)	Trimester 1 weight (kg)	Trimester 2 weight (kg)	Trimester 3 weight (kg)
15 – 19	Mean	17.98	163.03	62.60	69.780	75.29
	N	61	52	54	50	43
	Std. Deviation	1.18	5.87	15.12	15.5126	15.74
20 – 25	Mean	21.79	162.47	66.09	73.042	76.29
	N	96	78	88	73	66
	Std. Deviation	1.535	8.34	16.41	16.0908	15.92
Total	Mean	20.31	162.69	64.76	71.716	75.90
	N	157	130	142	123	109
	Std. Deviation	2.331	7.43	15.97	15.8758	15.78

### Haemoglobin pattern over the three trimesters

The results indicate that the haemoglobin concentration is highest in the first trimester, reaches its lowest point in the second trimester and begins to rise again in the third trimester (Table 2). The mean haemoglobin concentration was 12.73 ± 1.14 g/dl in the first trimester, 11.41 ± 1.16 g/dl in the second trimester and 11.67 ± 1.18 g/dl in the third trimester. These results are similar to those published by the Centers for Disease Control (CDC) using data from four European surveys of healthy women taking iron supplements [8] and the National Health and Nutrition Examination Survey (NHANES) [10].

**Table 2:** Mean (± S.D) haematological values over the three trimesters of pregnancy in primigravid women in the study population.

Parameters	Trimester 1	Trimester 2	Trimester 3	p-value		
				
				1^st ^& 2^nd^	1^st ^& 3^rd^	2^nd ^& 3^rd^
RBC (× 10^6^/μl)	4.33 ± 0.40	3.80 ± 0.33	3.94 ± 0.36	0.000*	0.054	0.000*
Hb (g/dl)	12.73 ± 1.14	11.41 ± 1.16	11.67 ± 1.18	0.000*	0.000*	0.009*
PCV (%)	37.05 ± 2.96	33.12 ± 3.00	34.03 ± 2.97	0.000*	0.000*	0.000*
MCH (pg)	29.53 ± 2.92	30.15 ± 3.01	29.85 ± 3.13	0.001*	0.000*	0.024*
MCHC (g/dl)	34.35 ± 1.05	34.42 ± 1.30	34.33 ± 1.43	0.753	0.457	0.373
MCV (fl)	85.89 ± 7.28	87.49 ± 7.02	86.70 ± 6.85	0.000*	0.819	0.001*
WBC (× 10^3^/μl)	8.27 ± 2.60	9.66 ± 2.84	8.79 ± 2.50	0.000*	0.187	0.000*
Platelets (× 10^3^/μl)	280.55 ± 64.40	250.32 ± 67.95	234.15 ± 67.67	0.000*	0.000*	0.000*

* indicates the level of significance p < 0.05.

Table legend text:

RBC – Red blood cell, Hb – Haemoglobin, PCV – Packed cell volume, MCH – Mean corpuscular haemoglobin, MCHC – Mean corpuscular haemoglobin concentration, MCV – Mean corpuscular volume, WBC – White blood cell

### Other haematological indices across trimesters

A similar trend in changes in concentration to that for the haemoglobin mentioned above, is also seen in the packed cell volume and the red blood cell count (Table 2). In contrast, the mean corpuscular volume and the mean cell haemoglobin had the lowest value in the first trimester, rose to its highest value in the second trimester and then started to decline in the third trimester. The mean corpuscular haemoglobin concentration however, remained fairly constant throughout pregnancy. The white blood cell count changed in a similar way as the mean corpuscular volume and the mean cell haemoglobin. The platelet count decreased from a mean value of 280.55 ± 64.40 × 10^3^/μl in the first trimester to 234.15 ± 67.67 × 10^3^/μl in the third trimester (Table 2).

### Comparisons across trimesters

The mean values for each variable (table 2) were compared between trimesters. The differences in the mean haemoglobin concentrations between the first and second trimesters, between the first and third trimesters and between the second and third trimesters were found to be statistically significant (p < 0.01). Similarly, the differences between the means across the three trimesters were statistically significant for the MCV (p < 0.01), WBC (p < 0.01) and the MCH (p < 0.01). A comparison of the means between the first and second trimester for the PCV was statistically significant (p = 0.001), but this was not so when the first and third or the second and third trimesters were compared. For the MCHC a statistically significant difference was observed only between the first and second trimester. In analyzing the red blood cell count, a statistically significant difference was observed between trimesters 1 and 3 and trimesters 2 and 3 while for the platelets the differences between the means observed across the three trimesters were not statistically significant.

A comparison of the 5^th ^percentile for the group with the quoted standards for haemoglobin concentration showed close correlation. For the 1^st ^trimester the 5^th ^percentile was 11.59 g/dl whereas the international standard is 11.0 g/dl. For the 2^nd ^trimester the 5^th ^percentile is 10.25 vs. 10.5 g/dl and for the 3^rd ^it is 10.5 vs. 11.0 g/dl.

## Discussion

As indicated in the introduction, haematological abnormalities, especially anaemia, may have an adverse impact on maternal and foetal well-being and pregnancy outcome. Significant effort is therefore given to monitoring and responding to haematological parameters. So far in most populations as in Jamaica this is based on international norms. From a search of the literature this appears to be the first attempt to compare haematological indices for anaemia in a Jamaican population with international norms and to determine their suitability as a standard for the population.

Haemoglobin concentrations generally remain stable until about the 16^th ^week of gestation after which there is a steady fall until it reaches its lowest point in the second trimester as a result of the expansion of the plasma volume. It is then expected that the haemoglobin concentration will remain constant or rise slightly during the third trimester when sufficient iron is available [2,4,8,11-13]. The results of this study are in keeping with expected trends and showed a decrease in haemoglobin concentration from the first to the second trimester followed by a slight increase in the third trimester. The results also indicate that the women maintained a good haemoglobin concentration throughout pregnancy as the mean values did not fall below the cut-off point for anaemia during any of the three trimesters. If the case definition for anaemia of <5^th ^percentile for the population was used the results would correlate fairly well with the current standards used to define anaemia across the populations. The changes in the red blood cell volume and packed cell volumes were similar to that in haemoglobin concentration in keeping with previous reports. The increase in these three parameters is a reflection of adequate iron supply resulting in increased haemoglobin production. However, the role of nutrition and or iron supplementation was not evaluated.

## Conclusion

In conclusion, the results of this first comprehensive study of the haematological profile of the Jamaican primigravida suggests that there is no significant difference in the haematological profile of the primigravid Jamaican woman when compared to international norms. This suggests that the international standards for anaemia in pregnancy are applicable to the Jamaican primigravid woman. This finding may be reassuring to others with a similar population and stage of development as Jamaica and suggest that they are applicable across populations.

## Competing interests

The author(s) declare that they have no competing interests.

## Authors' contributions

TJ was involved in the design of the study, carried out the data collection, analytical work, and analysis and was involved in drafting the manuscript. HR has made substantial contributions to the conception and design and coordination of the study as well as the critical review of the paper. AM has made substantial contributions to the conception, design and coordination of the study, as well as the drafting and critical revision of the paper.

All authors have read and approved the final manuscript.

## Pre-publication history

The pre-publication history for this paper can be accessed here:



## References

[B1] Focusing on anaemia: Towards an integrated approach for effective anaemia control. Joint statement by the World Health Organization and the United Nations Children's Fund. World Health Organization 2004. http://www.who.int/topics/anaemia/en/who.unicef-anaemiastatement.pdf.

[B2] Antenatal care: routine care for the pregnant woman. National Collaborating Centre for Women's and Children's Health Commissioned by the National Institute for Clinical Excellence Chapter 8, pg 67 – 68. RCOG Press, 27 Sussex Place, Regent's Park, London. http://www.rcog.org.uk/resources/public/pdf/Antenatal_care.pdf.

[B3] Klebanoff MA, Shiono PH, Selby JV, Trachtenberg AI, Graubard BI (1991). Anemia and spontaneous preterm birth. Am J Obstet Gynecol.

[B4] Allen LH (2000). Anemia and iron deficiency: effects on pregnancy outcome. Am J Clin Nutr.

[B5] Meng LZ, Goldenberg RL, Cliver S, Cutter G, Blankson M (1991). The relationship between maternal hematocrit and pregnancy outcome. Obstet Gynecol.

[B6] Reveiz L, Gyte GML, Cuervo LG Treatments for iron-deficiency anaemia in pregnancy. http://www.cochrane.org/reviews/en/ab003094.html.

[B7] Bothwell TH, Charlton RW (1981). Iron deficiency in women: a report of the International Nutritional Anemia Consultative Group (INACG).

[B8] Centers for Disease Control and Prevention (1998). Recommendations to prevent and control iron deficiency in the United States. MMWR Morb Mortal Wkly Rep.

[B9] Antenatal Care – 2.2 Pregnancy Complications; 2.2.1 – Anaemia, Pg 20. WHO/FRH/MSM/96.8, World health Organization 1996. http://www.who.int/reproductive-health/publications/MSM_96_8/MSM_96_8_chapter3.en.html.

[B10] (1993). Iron Deficiency Anemia; Recommended Guidelines for the Prevention, Detection, and Management Among U.S. Children and Women of Childbearing age. Institute of Medicine (IOM).

[B11] Yip R, PM Brook J, Halliday J, Powell L (1994). Changes in iron metabolism with age. Iron metabolism in health and disease.

[B12] Koller O (1982). The clinical significance of haemodilution during pregnancy. Obstet Gynecol Surv.

[B13] Anemia, From Wikipedia, the free encyclopedia. http://en.wikipedia.org/wiki/Anemia.

